# Internal supravesical hernia - a rare cause of intestinal obstruction: report of two cases

**Published:** 2012-01-26

**Authors:** Mahdi Bouassida, Selim Sassi, Hassen Touinsi, Helmi Kallel, Mohamed Mongi Mighri, Fathi Chebbi, Mechaal Ben Ali, Khaled Bouzeidi, Sadok Sassi

**Affiliations:** 1 Department of surgery, Mohamed Thahar Maamouri Hospital, Nabeul, Tunisia; 2 Department of radiology, Mohamed Thahar Maamouri Hospital, Nabeul, Tunisia; 3 Department of reanimation, Mohamed Thahar Maamouri Hospital, Nabeul, Tunisia

**Keywords:** Supravesical hernia, intestinal obstruction, surgery, Tunisia

## Abstract

Supravesical hernias develop at the supravesical fossa between the remnants of the urachus and the left or right umbilical artery. They are exceptional and are often the cause of intestinal obstruction. We report two cases of surgically proven internal supravesical hernias presenting with small bowel obstruction. Abdominal computed tomography showed, for our first case, the relation of the incarcerated intestine anterior to and compressing the urinary bladder. We believe that the preoperative diagnosis of supravesical hernia by abdominal computed tomography is possible, as shown in our first case.

## Introduction

Supravesical hernia is exceptional. It involves a hernia between the median umbilical ligament and the medial umbilical ligament and is classified as two types: internal supravesical hernia and external supravesical hernia. They are often the cause of intestinal obstruction. We report two cases of internal supravesical hernias presenting with small bowel obstruction. The first case was diagnosed preoperatively, but the second case was diagnosed intraoperatively.

## Observations

### Case 1

A 58-year-old woman with no relevant medical history was admitted with a one-day history of small bowel obstruction characterized by abdominal pain and vomiting. On examination, the patient was found in good general condition with a pulse rate of 80/ min, a blood pressure of 140/60 mmHg, and a temperature of 37.8°C. Physical and examination showed that the patient had abdominal distension without any peritoneal signs. Rectal examination was normal. Serum laboratory data were normal except for a white blood cell count of 12,400/ml.

**Figure 1:**
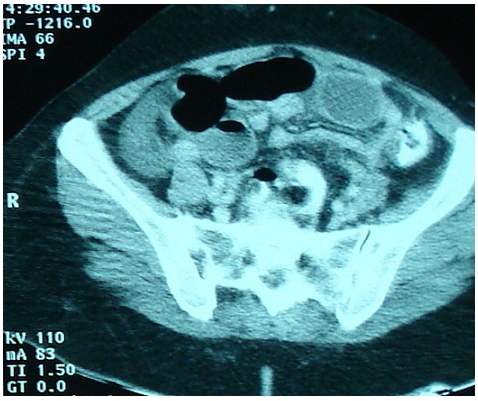
Computed tomography (CT) scan: a high-grade distal small bowel obstruction with a transitional zone in the left lower abdomen

**Figure 2:**
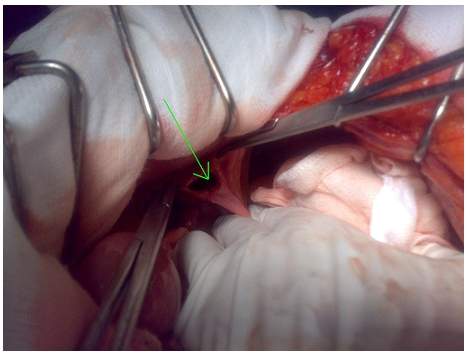
Operative view: defect in the pre-vesical fascia (arrow)

A scout film of the abdomen showed multiple dilated loops of small intestine with air–fluid levels. Computed tomography (CT) scan demonstrated a high-grade distal small bowel obstruction with a transitional zone in the left lower abdomen. Below the transitional zone, there was a saclike mass of clustered bowel loops within a hernia sac, indicating an internal hernia. The hernia sac was situated behind the left lower abdominal wall above the urinary bladder and passed downward into the prevesical space compressing the anterior bladder wall (Figure 1). Supravesical internal hernia was suggested. Exploratory laparotomy was done through midline incision after adequate fluid resuscitation. It revealed a loop of ileum herniating through a pouch in the supravesical fossa. The hernial ring was a 1-2 cm defect in the prevesical fascia. Digital exploration of defect after reduction of incarcerated bowel, revealed the sac to run medially and inferiorly to depress the wall of the bladder (Figure 2). The pregangrenous loop of ileum with doubtful viability was resected and an end-to-end anastomosis was performed. The pouch was closed with 1-0 prolene interrupted stitches. Post-operative period was uneventful and the patient was discharged on the post-operative day 11.

### Case 2

**Figure 3:**
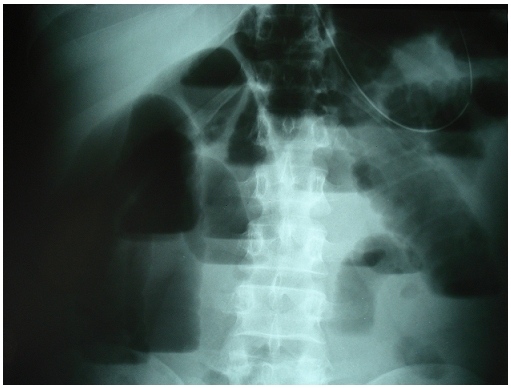
Scout film of the abdomen: multiple dilated loops of small intestine with air–fluid levels

A 36-year-old man was admitted to our hospital with symptoms of intestinal obstruction. He had experienced gradually increasing nausea, vomiting, and abdominal pain. He had no history of abdominal surgery. He had severe tenderness over the entire abdomen. Serum laboratory data were normal except for a white blood cell count of 14,500/ml. A scout film of the abdomen showed multiple dilated loops of small intestine with air–fluid levels (Figure 3).

Because of severe abdominal pain, he underwent emergency surgery with a lower midline abdominal incision. The intraabdominal findings included no ascites but a generally dilated intestine. Further exploration showed an ileal segment approximately 30 cm from the ileocecal valve that entered between the median and medial umbilical ligaments. A hernia loop consisting of 15 cm of the ileum was reduced with slight traction and was found to have simple obstruction with no impairment of the blood supply to the intestine. The hernia sac was 2 cm wide and 4 cm deep. It was located anterior to the bladder and posterior to the symphysis pubis, constituting an internal supravesical hernia. The opening of the sac was removed and closed with nonabsorbable sutures, and the intestine was then decompressed through a nasogastric tube. The patient's postoperative course was good, and he was discharged on postoperative day 8.

## Discussion

The supravesical fossa is the abdominal wall area between the remnants of the urachus (median umbilical ligament) and the left or right umbilical artery (medial umbilical ligament) [1,2]. The remnant of the urachus divides into the right and left fossa. There are two variants of supravesical hernias: an external form caused by the laxity of the vesical preperitoneal tissue, and an internal one with a growing hernia sac from back to front and above the bladder in a sagittal paramedian direction [1,3]. External supravesical hernia often occurs as a direct inguinal hernia. Except in specific cases of post-hernia surgery, supravesical hernias are almost always acquired and sometimes associated with inguinal hernias [1,3,4]. Skandalakis et al. [5] proposed the simpler terms “anterior supravesical”, “right or left lateral Supravesical”, and “posterior supravesical” depending on whether the hernia passed in front of, beside, or behind the bladder, respectively [4].

Pre-operative diagnosis of this condition is very difficult. In patients presenting with small bowel obstruction, without any history of previous abdominal operations and no obvious external hernias are detected, pre-operative investigations may be very helpful in diagnosing this condition [3,6]. A CT scan may suggest the diagnosis by showing the herniated loop so near the bladder that it actually distorts the wall[ [1,4]. Magnetic resonance imaging (MRI) and cystoscopy may also help in preoperative diagnosis [1], however, the majority of the recorded cases have been subjected to an exploratory laparotomy. The treatment is release of the intestinal obstruction and closing the hernial defect to prevent future recurrences. Most authors advise against attempts to excise the hernial sac and think that freshening the edges of the ring, with closure of the defect using continuous or interrupted stitches with nonabsorbable sutures is sufficient [3]. As some authors have reported, these procedures can be done via laparoscopy [7–9].

## Conclusion

Supravesical hernias are rare but potential causes of intestinal obstruction due to the confinement of loops in the supravesical fossa. CT findings have been reported in internal but not in external supravesical hernia. One of our cases of internal supravesical hernia indicate a possibility of preoperative diagnosis of supravesical hernia by abdominal CT.
